# Modulation of adhesion microenvironment using mesh substrates triggers self-organization and primordial germ cell-like differentiation in mouse ES cells

**DOI:** 10.1063/1.5072761

**Published:** 2019-03-22

**Authors:** Yuta Ando, Kennedy Omondi Okeyo, Taiji Adachi

**Affiliations:** 1 Department of Micro Engineering, Graduate School of Engineering, Kyoto University, Kyoto daigaku-katsura, Nishikyo-ku, Kyoto 615-8530, Japan; 2 Institute for Frontier Life and Medical Sciences, Kyoto University, 53 Shogoin Kawahara-cho, Sakyo-ku, Kyoto 606-8507, Japan; 3 Division of Systemic Life Science, Graduate School of Biostudies, Kyoto University, Yoshida-Konoecho, Sakyo-ku, Kyoto 606-8501, Japan

## Abstract

The cell adhesion microenvironment plays contributory roles in the induction of self-organized tissue formation and differentiation of pluripotent stem cells (PSCs). However, physical factors emanating from the adhesion microenvironment have been less investigated largely in part due to overreliance on biochemical approaches utilizing cytokines to drive *in vitro* developmental processes. Here, we report that a mesh culture technique can potentially induce mouse embryonic stem cells (mESCs) to self-organize and differentiate into cells expressing key signatures of primordial germ cells (PGCs) even with pluripotency maintained in the culture medium. Intriguingly, mESCs cultured on mesh substrates consisting of thin (5 *μ*m-wide) strands and considerably large (200 *μ*m-wide) openings which were set suspended in order to minimize the cell-substrate adhesion area, self-organized into cell sheets relying solely on cell-cell interactions to fill the large mesh openings by Day 2, and further into dome-shaped features around Day 6. Characterization using microarray analysis and immunofluorescence microscopy revealed that sheet-forming cells exhibited differential gene expressions related to PGCs as early as Day 2, but not other lineages such as epiblast, primitive endoderm, and trophectoderm, implying that the initial interaction with the mesh microenvironment and subsequent self-organization into cells sheets might have triggered PGC-like differentiation to occur differently from the previously reported pathway via epiblast-like differentiation. Overall, considering that the observed differentiation occurred without addition of known biochemical inducers, this study highlights that bioengineering techniques for modulating the adhesion microenvironment alone can be harnessed to coax PSCs to self-organize and differentiate, in this case, to a PGC-like state.

## INTRODUCTION

Pluripotent stem cells (PSCs) can differentiate to nearly all cell lineages and are capable of self-organization to generate multicellular tissues, for instance organoids.^1^ To induce PSCs to self-organize, recent research studies have been focusing on replicating *in vivo* conditions using three-dimensional culture systems in combination with biochemical factors, such as cytokines, to induce specific differentiation and tissue formation.^2–4^ Indeed, a number of studies have reported that mechanical and geometrical factors on fabricated culture substrates, such as substrate stiffness, surface topography or micropattern, could trigger self-organization and differentiation through cell adhesion and cell-cell interaction.^5–7^ These studies show that the emergence of ordered germ layers and/or self-organized structures from a population of PSCs is governed by mechanical and geometrical factors as well as biochemical factors in the extracellular microenvironment. Hence, bioengineering techniques for designing the physical microenvironment will provide a powerful approach to drive the intrinsic self-organization property of cells.

Here, we have developed a culture method to drive PSC self-organization and differentiation by modulating the cell adhesion microenvironment using microstructured mesh substrates.^8^ The underlying hypothesis is that PSC self-organization can be induced by mechanical and geometrical factors inherent in the adhesion microenvironment through two types of cell adhesions: cell-substrate and cell-cell adhesion. In fact, previously, by culturing human induced pluripotent stem cells (hiPSCs) on suspended mesh sheets with large openings (>100 *μ*m) and narrow strands (5 *μ*m in width) to restrict the cell-substrate adhesion area, we demonstrated that these cells can form self-organized cysts exhibiting trophectoderm-like features.^9,10^ These studies demonstrated that controlling the adhesion microenvironment is a plausible strategy to recapitulate events of early embryogenesis by hiPSCs, but the mechanism is still unclear.

Primordial germ cells (PGCs), the first reproductive lineage cells, originate from proximal cells of early post implantation epiblast at the stage of E6.0 egg cylinder during mouse early embryo development.^11^ Given the difficulty of *in vivo* investigation of human PGC development due to ethical issues, PGC derivation from PSCs is a hot topic in medical and developmental research fields because the process will contribute toward understanding PGC specification, which remains less understood. Indeed, previous and ongoing research studies have already established induction protocols for generating PGC-like cells from mouse and human PSCs using cytokine stimulation.^12,13^ However, biochemical-based approaches cannot capture the full landscape of PGC development, in particular, the roles played by physical factors resulting from the interaction between cells and the physical microenvironment. In fact, it is well known that the physical microenvironment plays important roles in cell fate decision making during *in vivo* mouse embryo development,^14,15^ although this is less investigated in the case of PGC specification. Thus, a bioengineering approach for elucidating the role of the physical microenvironment on PGC development is highly desirable, but to the best of our knowledge, no such approach has been reported in the literature.

In this study, we demonstrate that the modulating cell adhesion microenvironment alone can trigger self-organization and differentiation to a PGC-like state. Specifically, mouse embryonic stem cells (mESCs) cultured on microstructured mesh substrates exhibited self-organization into cell sheets by Day 2 and, subsequently, into dome-shaped cysts at around Day 6. Importantly, examination of sheet-forming cells revealed differential expressions of PGC-related genes as early as Day 2 of mesh culture. Given that we did not carry out any biochemical stimulations, i.e., no addition of typically used cytokines, we postulate that the observed spontaneous differentiation to PGC-like cells is an attribute of cell-cell interaction with the mesh-defined adhesion microenvironment. Thus, our study provides an alternative hitherto less investigated approach for the derivation of PGC-like differentiation using microstructured cell culture substrates.

## RESULTS

### mESCs self-organized under adhesion restriction on a mesh substrate

**FIG. 1.**
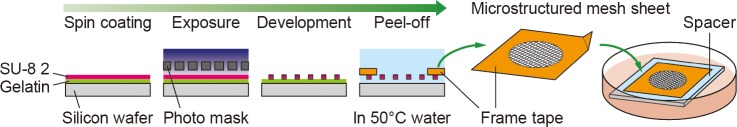
Schematic illustration of the mesh fabrication and setup for cell culture. Microstructured mesh sheets were fabricated by photolithography using SU-8 2. Mesh sheets were reinforced with frame tapes with a punched hole (4 mm in diameter) and then the setup was suspended on a culture dish using a 0.5 mm-thick silicon rubber spacer with a punched hole (6 mm in diameter).

**FIG. 2.**
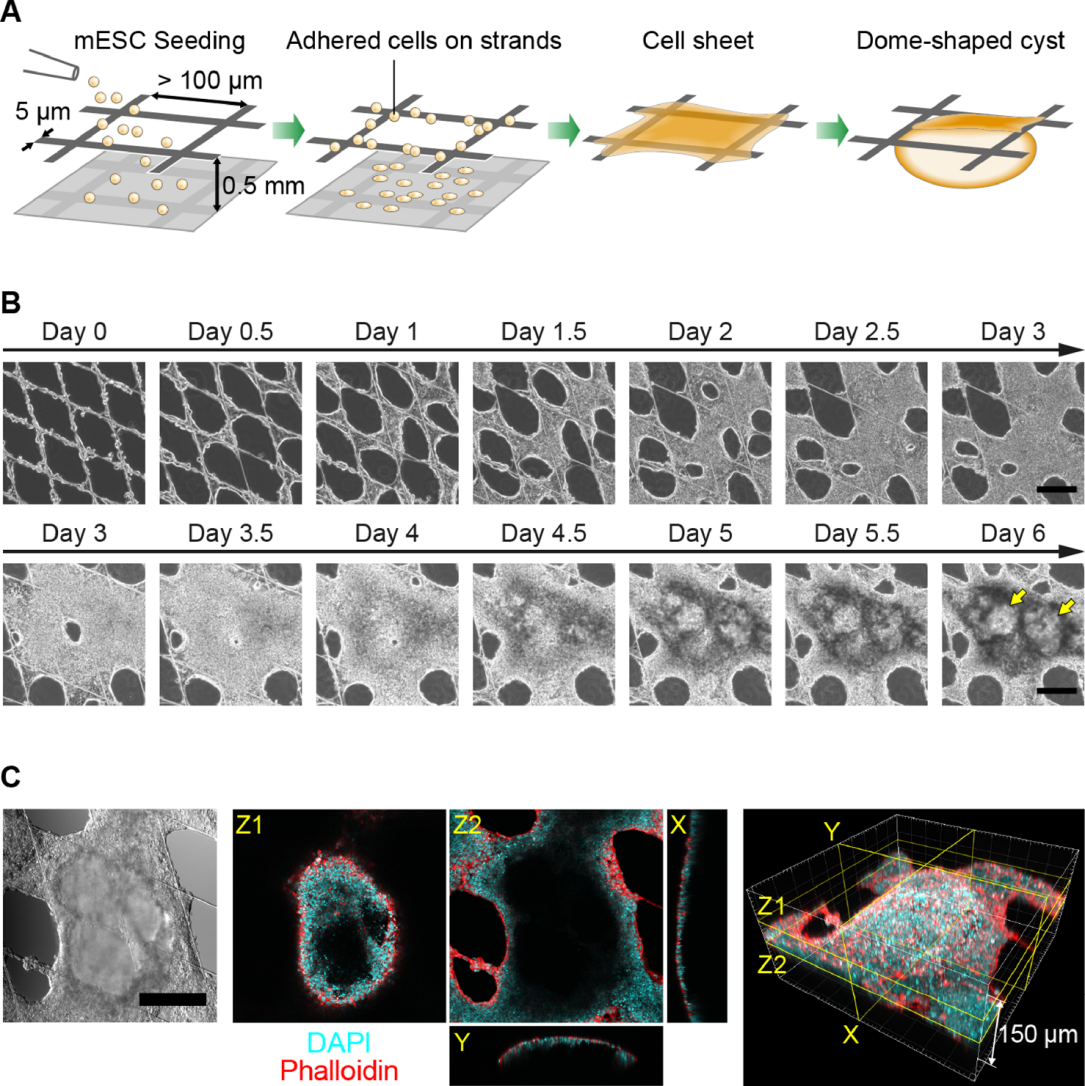
Self-organization of mESCs cultured on microstructured mesh sheets. (a) Schematic illustration of mESC culture on mesh sheets. mESCs cultured on the suspended mesh sheets self-organized to generate cell sheets and dome-shaped cysts. (b) Timelapse images of mesh-cultured mESCs from Day 0 to Day 3 and from Day 3 to Day 6, taken separately. Cells on the mesh strands proliferated successfully and began to fill the mesh openings to form cell sheets by Day 3. Between Day 4 and Day 6, dorm-shaped cyst formation occurred on the cell sheets spontaneously. The yellow arrows on the image at Day 6 indicate the generated dome-shaped cysts. Scale bars: 200 *μ*m. (c) Confocal and 3D reconstructed images of a dome-shaped cyst at Day 6. DAPI (cyan) stained nucleus and Phalloidin (red) stained F-actin. Scale bar: 200 *μ*m.

To modulate a cell adhesion microenvironment, we fabricated microstructured mesh sheets with narrow mesh strands (5 *μ*m in width) and large mesh openings (rhombus shape with a minor axis of 200 *μ*m), which were then setup suspended on a culture dish and used as substrates for mESC culture (Fig. 1). To monitor cell behavior on these culture substrates, we performed live cell imaging of mESCs cultured in the culture medium with leukemia inhibitory factor (LIF). A schematic illustration of cell dynamics on the mesh sheets is shown in Fig. 2(a). Seeded mESCs attached to the narrow mesh strands, underwent proliferation and self-organized to form cell sheets over the mesh openings by Day 2 [Fig. 2(b), Days 0–3 and supplementary material, Movie 1]. Remarkably, since the mesh sheets are suspended, the closing of the interior of mesh openings occurred without cell-substrate adhesion, implying that cells relied on cell-cell interaction to self-organize, resulting in the formation of cell sheets over the initially empty mesh openings. With continued culture under phase contrast microscopy, we observed a gradual darkening of the already formed cell sheets, suggesting a gradual increase in the sheet thickness [Fig. 2(b)]. Intriguingly, between Day 4 and Day 6, dome-shaped cysts emerged from the cell sheets [Fig. 2(b), yellow arrows at Day 6 and supplementary material, movie 2]. These cysts were observed mostly on the lower side of the mesh sheets and were typically 200–400 *μ*m in diameter and 50–200 *μ*m in height. 3D reconstruction of images acquired by immunofluorescence microscopy showed that the cysts were hollow and had a thin wall which was several cells in thickness, and exhibited an F-actin cortex, which appeared more enriched on the outside than the inside of the wall [Fig. 2(c)].

### Microarray analysis revealed PGC-like differentiation by mesh culture

Next, to characterize the observed self-organization, we examined gene expression profiles at different stages of mesh culture using microarray analysis. For this purpose, we used mesh-cultured cells at Day 2, Day 3 and Day 6, with dish-cultured mESCs at Day 2 serving as controls. Here, mesh-cultured cells at Day 2 represented the early first phase of self-organization leading to cell sheet formation, cells at Day 3 represented late phase of sheet formation, and cells at Day 6 represented the second phase of self-organization into dome-shaped cysts. A pairwise comparison of top-50 up-regulated and down-regulated genes selected on the basis of fold-change is shown in supplementary material, Tables I and II. Expression results of the four samples were subjected to one-way analysis of variance (ANOVA) test (P-value < 0.00001) to select 1287 genes, which were then categorized into 8 clusters by hierarchical clustering analysis (Pearson correlation and average linkage, distance threshold = 0.35). As shown in supplementary material, Suppl. Fig. 1(a), clusters I–V and VI–VIII were mostly up-regulated and down-regulated gene sets, respectively, during the mesh culture. Notably, gene ontology (GO) enrichment analysis showed that terms related to developmental processes and reproduction (such as “GO:0050793 ∼ regulation of the developmental process,” “GO:0003006 ∼ developmental process involved in reproduction” and “GO:0022414 ∼ reproductive process”) appeared in clusters I–IV (supplementary material, Table III), which contain up-regulated genes, indicating that these gene terms were modulated by the mesh culture.

**FIG. 3.**
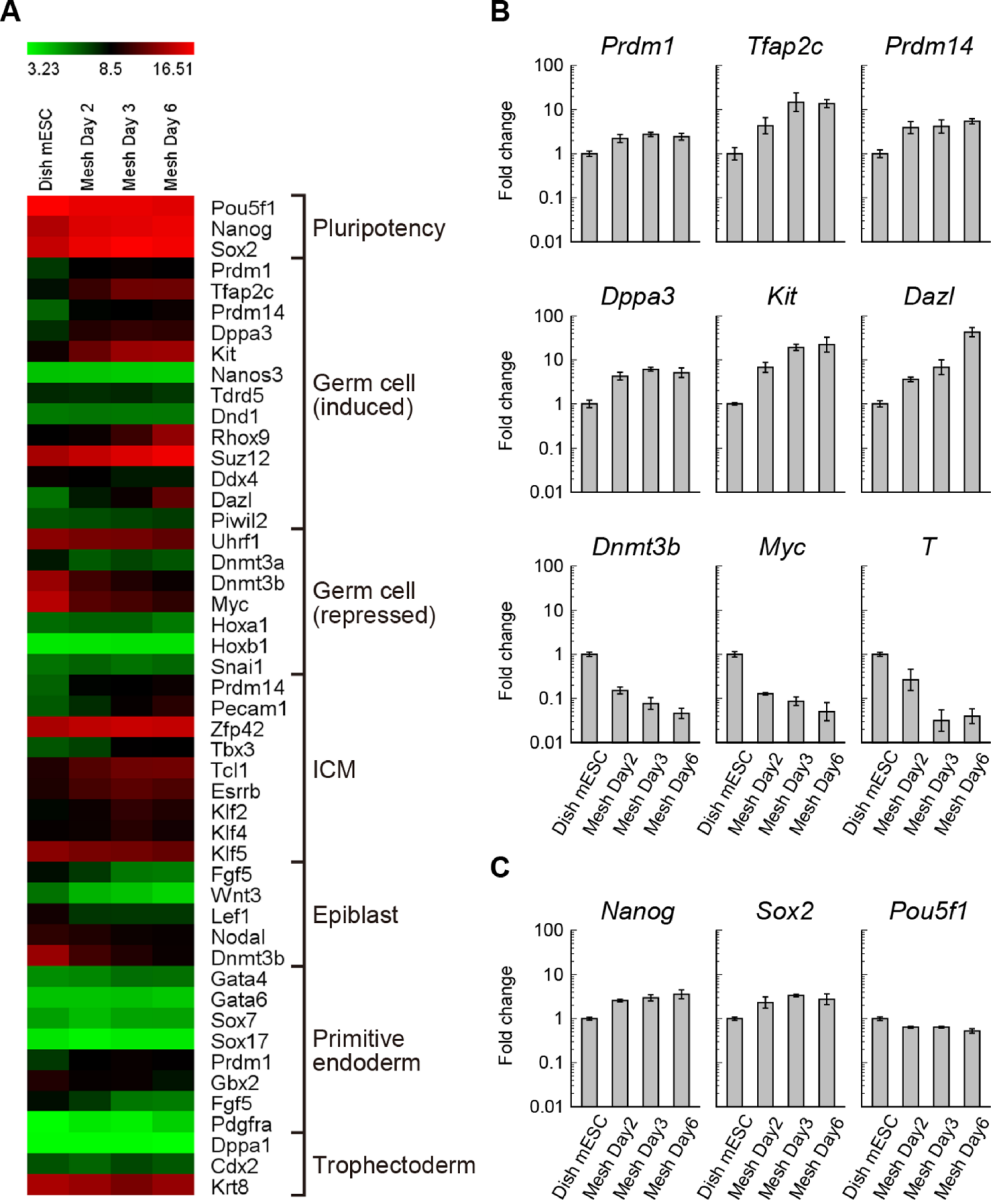
Differential expression analysis of microarray data for PGC-related genes from mesh-cultured cells at Day 2 (n = 3), Day 3 (n = 3), Day 6 (n = 4), and from dish-cultured mESCs (n = 3) as control. (a) Heatmap display of genes related to germ cell and early embryogenesis. Germ cell markers were selected on the basis of previous studies in Refs. 12, 16, and 17, while ICM, epiblast, primitive endoderm, and trophoectoderm markers were selected on the basis of previous studies in Refs. 12, 18, and 19. (b) Fold changes of PGC marker genes (*Prdm1*, *Tfap2c*, *Prdm14*, *Dppa3*, *Kit*, and *Dazl*) in the log10 scale, with SDs. (c) Fold changes of mESC pluripotency marker genes (*Nanog*, *Sox2* and *Pou5f1*) in the log10 scale, with SDs.

Prompted by the results of GO enrichment analysis, we further analyzed lineage markers associated with during early mouse embryo development in order to identify the differentiated cell lineage. For this purpose, we focused on the period between the E3.5 blastocyst and E6.0 egg cylinder stages, which correspond to stages between the emergences of the inner cell mass (ICM, the origin of mESCs) and PGCs (the first reproductive lineage). As shown in Fig. 3(a), we found differential expression changes for several genes associated with germ cell specification, including the core genes of the PGC transcription network such as BLIMP1 (encoded by *Prdm1*), AP-2γ (encoded by *Tfap2c/Tcfap2c*), and PRDM14.^16^ Based on earlier reports about PGC-like differentiation from PSCs,^12,16,17^ we selected gene lists and evaluated the differential expression changes. Our study shows that, in particular, *Prdm1*, *Tfap2c*, *Prdm14*, *Dppa3/Stella/Pgc7*, *Kit*, and *Dazl* were statistically up-regulated (P-value < 0.00002), illustrating the possibility of mouse PGC-like differentiation by the mesh-cultured mESCs, consistent with previous reports. Indeed, genes related to PGC specification such as *Tfap2c*, *Kit*, and *Dazl* showed more than 10-fold change [Fig. 3(b)]. Consistently, *Dnmt3b*, *Myc*, and *T*, which are repressed in PGCs, showed less than 0.1-fold change [Fig. 3(b)]. However, *Nanos3* and *Dnd1* were lowly expressed, inconsistent with the result of the previous *in vitro* PGC induction method.^12,17^ Among the mESC pluripotency marker genes, except *Pou5f1* (encoding OCT3/4) which was not statistically changed, *Nanog* and *Sox2* were up-regulated [Figs. 3(a) and 3(c)]. The fact that these pluripotency markers kept high expression levels under the mesh culture was consistent with the expression of pluripotency markers in PGC-like cells.^12^ Moreover, consistent with these observations, the expression of epiblast, primitive endoderm and trophectoderm markers^12,18,19^ was mostly repressed [Fig. 3(a)]. Furthermore, most of the master regulator genes associated with three primary germ layers^20^ were lowly expressed in the mesh-cultured cells [supplementary material, Suppl. Fig. 1(b)]. Taken together, these results rule out the possibility of aberrant differentiation and support the possibility that the mesh culture triggered the differentiation of mESCs to the PGC-like state.

### Immunofluorescence microscopy confirmed the expression of PGC markers

**FIG. 4.**
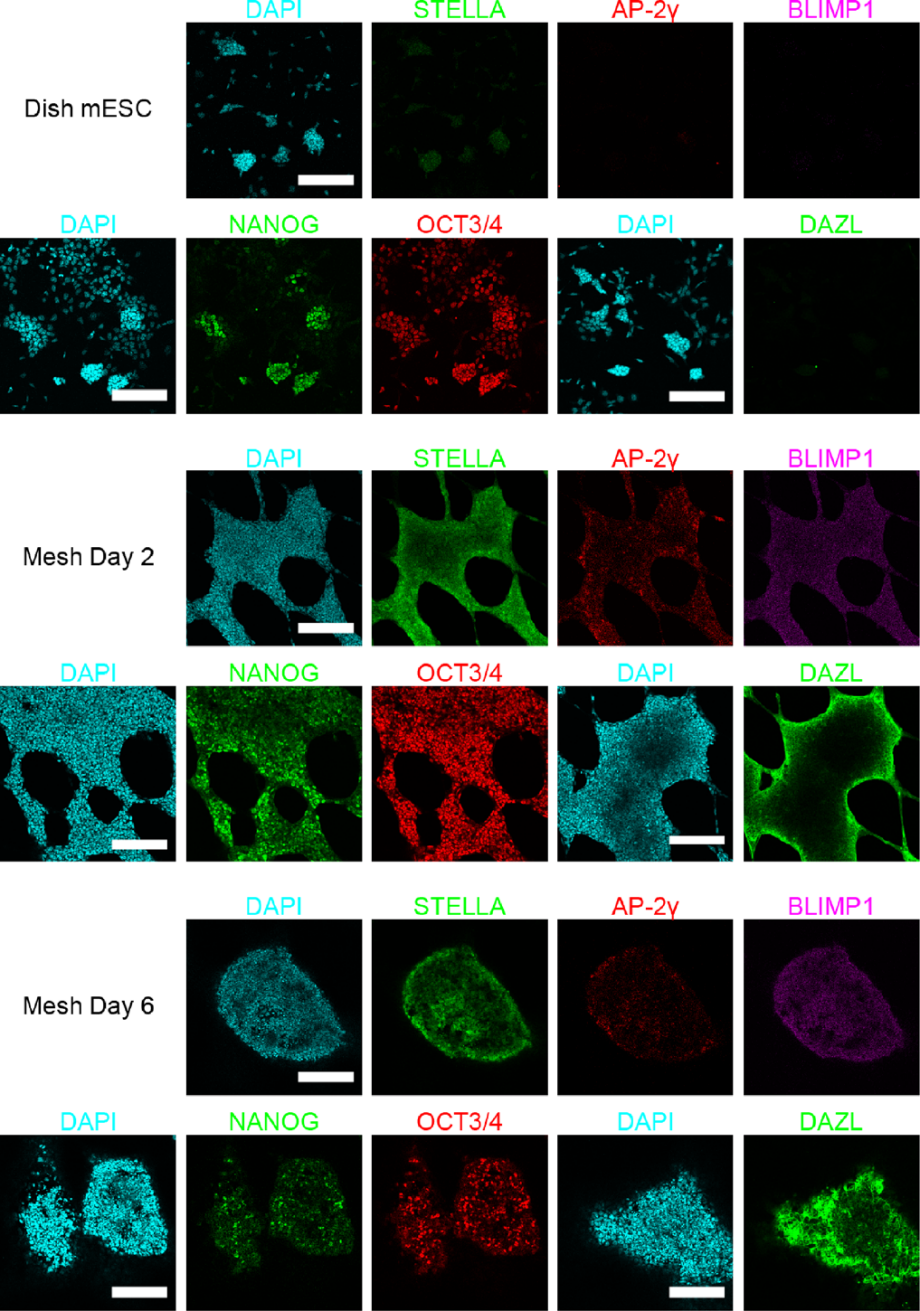
Comparative expression of PGC and pluripotency markers in dish-cultured mESCs versus mesh-cultured cells at Day 2 and Day 6 obtained by confocal microscopy. We performed immunofluorescence staining for STELLA (green), AP-2γ (red), BLIMP1 (purple), DAZL (green), NANOG (green), and OCT3/4 (red). For each sample, co-staining was performed for STELLA, AP-2γ, and BLIMP1, and separately for NANOG and OCT3/4. Scale bars: 200 *μ*m.

To further examine the expression of PGC marker proteins, we performed immunofluorescence microscopy on dish-cultured control mESCs and mesh-cultured cells at Day 2 and Day 6 (Fig. 4). We stained for STELLA, AP-2γ, BLIMP1, and DAZL; proteins coded by the major PGC genes, *Dppa3*, *Tfap2c*, *Prdm1*, and *Dazl* whose statistical up-regulation was confirmed by microarray analysis. In addition, the two major pluripotency markers, NANOG and OCT3/4, were also immunostained. Although some mesh-cultured cells showed reduced expression of NANOG and OCT3/4, a visual inspection of the immunofluorescence results shows that there were cells that highly expressed these two proteins at Day 6. Since microarray analysis tends to be sensitive to high expression cells, we believe that the microarray and immunofluorescence results are consistent. Interestingly, STELLA, which appeared to be weakly expressed in dish-cultured mESCs, was relatively strongly expressed in mesh-cultured cells at Day 2 and Day 6. In addition, compared with diminished expression in control mESCs, mesh-cultured cells at Day 2 and Day 6 showed relatively higher expression of AP-2γ, with AP-2γ positive cells mostly localized at the periphery of the cell population. Furthermore, BLIMP1 was weakly detected in mesh-cultured cells at Day 2 and Day 6 but not in dish-cultured mESCs. However, the localization pattern observed in this study was rather global, which is different from the nuclear localization reported in previous research.^21^ Importantly, DAZL showed high-level expression in mesh-cultured cells at Day 2 and Day 6, especially in the periphery of the cell population, further supporting PGC-like differentiation.

Since the formation of dome-shaped cysts was observed by Day 6, we were interested in investigating PGC marker expression in the cysts. Immunofluorescence microscopy at Day 6 revealed that the expression of PGC markers outlined above did not show specific spatial distribution in the dome-shaped cysts (Fig. 4 and supplementary material, Suppl. Fig. 2). This indicates that the formation of dome-shaped cysts and PGC-like differentiation have little correlation, in accordance with the results of our microarray analysis which show that PGC-like differentiation begins as early as from Day 2 of mesh culture (Fig. 3).

## DISCUSSION

We have shown that mESCs cultured on microstuctured mesh sheets proliferated, overcame the substrate restriction relying on cell-cell interaction, filled the mesh openings, and formed the cell sheets by Day 3 [Fig. 2(b)]. From the self-organized cell sheets, dome-shaped cysts characterized by thin wall emerged between Day 4 and Day 6 [Figs. 2(b) and 2(c)]. These cysts exhibited a rich band of F-actin on the outside of the enclosing wall [Fig. 2(c)], suggesting an inward-outward polarity which would be important for the formation of a cystic structure. Based on the microarray result [supplementary material, Suppl. Fig. 3(a)], the expression of genes associated with extracellular matrixes (ECMs) such as fibronectin 1, collagen type I α2, type IV α2, laminin α1, and β2 were up-regulated in mesh-cultured cells. This may suggest that mesh-cultured cells secret ECMs where cells can attach to overcome the mesh openings and which can affect the inward-outward polarity, but more investigations are required to confirm the mechanisms.

In our study, up-regulation of PGC markers was confirmed using microarray analysis and immunofluorescence microscopy in mESCs cultured on mesh substrates with an LIF-supplemented culture medium. Although some PGC marker genes such as *Prdm1*, *Tfap2c*, *Dppa3*, and *Dazl* can be expressed in mESCs, as mentioned in previous studies,^18,20,22^ our study shows that all of these gene expressions were up-regulated in mesh-cultured cells compared with dish-cultured mESCs. This clearly shows that the mesh culture environment triggered the shift to the PGC-like state. Earlier studies reported that LIN28 suppresses let-7 miRNA maturation and that let-7 miRNAs are up-regulated only during the later stages of PGC development.^23,24^ Based on the microarray analysis, although *Lin28a* was down-regulated during mesh culture, *Nanos3*, an important gene of PGC development,^25^ was not up-regulated in our result, making it difficult to determine conclusively the differentiation stage.

The biochemical mechanism of PGC development *in vivo* is already known, i.e., PGCs appear in proximal cells of early post implantation epiblast in response to bone morphogenetic protein 4 (BMP4) from the extra-embryonic ectoderm and BMP2 from the visceral endoderm.^16^ To recapitulate PGC development, previous methods have established two-steps for *in vitro* derivation of PGC-like cells: naïve mESCs under the two inhibitor (2i) condition^26^ are primed to epiblast-like cells and then to PGC-like cells.^12^ In this PGC derivation method, the marker genes of epiblast and mesoderm; *Dnmt3b* and *T*, are up-regulated transiently, whereas the pluripotency markers; *Nanog* and *Sox2*, are repressed in epiblast-like cells, which are in the intermediate state of differentiation to PGCs.^12^ On the contrary, our microarray result shows that while pluripotency markers maintained a high-level expression during the 6-Day analysis period, and other lineage markers related to epiblast, primitive endoderm and trophectoderm were repressed [Fig. 3(a)]. In addition, based on the comparison of the PGC-related gene expression pattern with the previous induction method,^12^ the pattern of gene expression changes in this study was consistent with the pattern from epiblast-like cells to PGC-like cells, not from mESCs to epiblast-like cells reported in the previous study (supplementary material, Table IV). In particular, the known PGC-related genes, such as *Prdm1*, *Tfap2c*, *Prdm14*, *Dppa3*, *Kit*, *Dazl*, *Dnmt3a*, *Dnmt3b*, and *Myc*, were changed under the mesh culture, consistent with the observed PGC-like differentiation in the previous study. In addition, integrin β3, a surface marker of PGC-like cells,^12^ was up-regulated by the mesh substrates (supplementary material, Suppl. Fig. 3). Although the exact mechanism remains to be elucidated, these results suggest that the mesh culture may derive PGC-like differentiation via a pathway independent of the conventional naïve mESCs to epiblast-like differentiation.

**FIG. 5.**
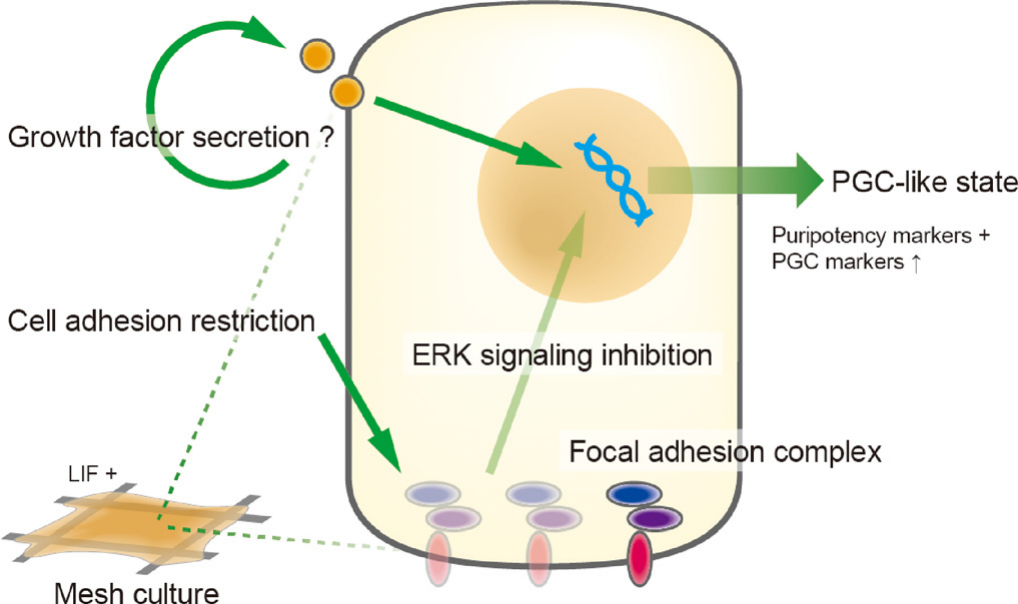
A plausible mechanism for PGC-like differentiation under the mesh culture. We hypothesize that the mesh culture microenvironment may act as a primary trigger of ERK signaling inhibition via restriction of the formation of focal adhesion complexes and growth factor secretion which acts to eventually initiate PGC-like differentiation.

A plausible mechanism for the PGC-like differentiation observed in our study can be argued on the basis of a previous report that have demonstrated that extracellular signal-regulated kinase (ERK) signaling inhibition induces up-regulation of PGC marker genes in mESCs under mesodermal differentiation condition using OP9 feeder cells.^17^ Indeed, it has been reported that ERK signaling pathway can be responsive to components of the focal adhesion complex.^27^ In this scenario, because the mesh substrates restrict the cell-substrate adhesion area, it is likely that inadequate formation of focal adhesion complexes may repress ERK signaling pathways, leading to a PGC-like state via intracellular signaling. This is supported by the microarray results of genes related to cell adhesion complexes and ECMs as shown in supplementary material, Suppl. Fig. 3, i.e., the expression of vinculin, talin 1, integrin α3, and α5 genes was down-regulated after cell seeding to mesh substrates. Another notable observation was that mesodermal marker genes (*Hoxa1*, *Hoxb1*, and *Snai1*) remained repressed (supplementary material, Suppl. Fig. 1), whereas *Vegfa* was highly up-regulated during the mesh culture. In the context of earlier studies using OP feeder cells which demonstrated that ERK signaling inhibition may antagonize mesodermal differentiation and promote PGC specification,^17^ other factors secreted by OP9 feeder cells, for example, vascular endothelial growth factor (VEGF), also may be important in PGC specification. In this respect, we hypothesize that the mesh culture microenvironment may act as a primary trigger of ERK signaling inhibition via restriction of the formation of focal adhesion complexes and growth factor secretion which acts to eventually initiate PGC-like differentiation (Fig. 5). The full mechanism of this landscape will be explored in our future studies focusing on mechanotransduction signaling pathways related to ERK signaling.

## CONCLUSION

Our study shows that restricting the cell adhesion area by our original mesh culture system can induce mESCs to self-organize into cell sheets and then dome-shaped cysts. Remarkably, the self-organization processes triggered by the mesh microenvironment accompanied PGC-like differentiation, as evidenced by the expression of key PGC signatures. Interestingly, the observed PGC-like differentiation occurred without addition of biochemical PGC differentiation inducers such as cytokines, but solely by modulating the cell adhesion microenvironment using the mesh substrates. Hence, it can be argued that physical factors attributable to the mesh microenvironment may have contributed to the self-organization and PGC-like development of mESC through intrinsic factors associated with cell adhesion, such as ERK signaling pathway suppression. However, the relationship between dome-shaped cyst formation and PGC-like differentiation is still unclear, and the exact mechanism through which these events are triggered remains to be clarified. To suffice, contrary to contemporary studies which rely largely on biochemical factors to induce self-organized tissue formation and differentiation by PSCs, our mesh culture system can be viewed as a biophysical inducer of self-organization by restricting the cell-substrate adhesion area.

## METHODS

### Cell culture

In this study, we used mESC line E14tg2a (Riken Cell Bank, Japan).^28^ mESCs were cultured at 37 °C and 5% CO_2_ on cell culture dishes coated with 0.1% gelatin from porcine skin (gel strength 300, type A; Sigma-Aldrich, USA). The cells were maintained with Glasgow Minimum Essential Medium (G-MEM; Wako, Japan) with 15% fetal bovine serum (FBS) (Sigma-Aldrich), 1 mmol/L sodium pyruvate (Wako), 1% MEM non-essential amino acids (Wako), 0.1 mmol/l 2-mercaptoethanol (Wako), and 1000 units/ml LIF (Wako). The cells were dissociated by TrypLE Express (Thermo Fisher Scientific, USA) and passaged at 7.0 × 10^3^ cells/cm^2^ for 2-Day interval passage or 2.6 × 10^3^ cells/cm^2^ for 3-Day interval passage.

### Fabrication and setup of microstructured mesh sheets

Microstructured mesh sheets were fabricated by photolithography using SU-8 2 (MicroChem, USA), an epoxy-based negative photoresist, as reported previously.^8^ A schematic illustration of the procedures is given in Fig. 1. Briefly, first, pre-warmed 2% gelatin from bovine skin (SAJ special grade; Sigma-Aldrich) was spin-coated on a silicon wafer at 2000 rpm. Gelatin coating formed a sacrificial layer for peeling-off the mesh sheets. Then SU-8 2 was spin-coated at 2000 rpm to form a 2 *μ*m-thick layer and the wafer was baked at 65 °C for 1 min and 95 °C for 3 min, respectively. The wafer was exposed to 10 mW/cm^2^ ultraviolet light for 12 sec using designed photomasks and baked at 65 °C for 1 min and 95 °C for 1 min. Pattern development was performed in propylene glycol methyl ether acetate (PGMEA) and then isopropanol. The mesh pattern was reinforced by laminating with a Kapton polyimide film (Nitto, Japan) with a punched hole (4 mm in diameter) and was peeled off in a 50 °C water bath. Finally, the mesh sheet was picked up and mounted on a 0.5 mm-thick silicon rubber spacer with a punched hole (6 mm in diameter). After fabrication, the sterilization of mesh sheets was performed under a ultraviolet lamp for 12 h. In this study, we used mesh sheets with rhombus-shaped openings (minor axis 200 *μ*m, apex angle 50°) intercalated with narrow strands (5 *μ*m in width).

### Seeding and culture of mESCs on mesh sheets

Protein coating to support cell attachment on the mesh strands was performed before cell seeding using 10 *μ*g/ml laminin-511 E8 fragment (iMatrix-511; Nippi, Japan) solution in phosphate buffered saline (PBS) for 12 h at 4 °C. Using the typical cell passaging protocol, the concentration of the mESC suspension was adjusted to 4.0–8.0 × 10^6^ cells/ml for seeding on the mesh sheets. Then, 100 *μ*l of the suspension were added on mesh sheets gently, after which, cells were incubated for 6 h at 37 °C and 5% CO_2_ to allow cell attachment on the mesh strands. Then, the cell-seeded mesh sheets were transferred to a new culture dish in order to discard fallen cells. Finally, fresh culture medium was added and culture continued undisturbed. Day 0 was defined as the start of cell culture on mesh sheets. Culture medium was exchanged after every 3 days.

### Live cell imaging and immunofluorescence microscopy

For live cell imaging to capture cell dynamics on the mesh sheets, we performed timelapse microscopy using a BZ-X700 fluorescent microscope (Keyence, Japan) equipped with an INUG2-KIW stage-top incubator (Tokai hit, Japan).

For immunofluorescence microscopy, cells were fixed by 4% paraformaldehyde for 30 min at room temperature and permeabilized with 0.1% TritonX-100 (MP Biomedicals, USA) for 15 min at room temperature. After blocking with 3% bovine serum albumin (Sigma-Aldrich) for 30 min at room temperature, primary and secondary antibodies diluted in 1% bovine serum albumin were added and incubated overnight at 4 °C. For primary antibodies, the STELLA antibody (1:200; ab19878; Abcam, UK), AP-2γ antibody (1:100; sc-12762; Santa Cruz Biotechnology, USA), Blimp1 antibody (1:50; 14–5963-80; Thermo Fisher Scientific), DAZL antibody (1:200; ab34139; Abcam), OCT-3/4 antibody (1:100; sc-5279; Santa Cruz Biotechnology), and NANOG antibody (1:200; ab80892; Abcam) were used. The corresponding secondary antibodies used were the F(ab′)2-goat anti-Mouse IgG (H+L) cross-adsorbed secondary antibody (1:500; A-11017 or A-11018; Thermo Fisher Scientific), F(ab′)2-goat anti-Rabbit IgG (H + L) cross-adsorbed secondary antibody (1:500; A-11070 or A-11071; Thermo Fisher Scientific), and Goat anti-Rat IgG (H + L) Cross-Adsorbed Secondary Antibody (1:500; A-21247; Thermo Fisher Scientific). 4′,6-diamidino-2-phenylindole (DAPI; 1:500; D1306; Thermo Fisher Scientific) and Alexa Flour 546 Phalloidin (1:30; A22283; Thermo Fisher Scientific) were used for staining the cell nucleus and F-actin cytoskeleton, respectively. Immunofluorescence microscopy was performed with a FLUOVIEW FV3000 confocal laser scanning microscope (Olympus, Japan). The acquired images were processed and reconstructed using ImageJ (National Institutes of Health, USA) and Imaris (Bitplane, Switzerland).

### RNA isolation and microarray analysis

RNA samples were collected using ISOGEN (Nippon Gene, Japan), chloroform, and isopropanol and purified by NucleoSpin RNA (Macherey-Nagel, Germany) according to the manufacturer's instructions manuals. Microarray analysis of RNA samples from dish-cultured mESCs (n = 3), mesh-cultured cells at Day 2 (n = 3), Day 3 (n = 3) and Day 6 (n = 4) was performed using Clariom S Assay for mouse (Thermo Fisher Scientific). For data analysis, we used the Transcriptome Analysis Console (TAC) 4.0 (Thermo Fisher Scientific). Gene ontology (GO) enrichment analysis was performed by the DAVID Bioinformatics Resources 6.8 (https://david.ncifcrf.gov/).^29–31^ Hierarchical clustering analysis and heatmap drawing were performed by the Multiple Experiment Viewer (http://mev.tm4.org).

### Ethics approval

Ethics approval is not required for this study.

## SUPPLEMENTARY MATERIAL

See supplementary material for additional experimental data and information.
